# Reduced Stress and Improved Sleep Quality Caused by Green Tea Are Associated with a Reduced Caffeine Content

**DOI:** 10.3390/nu9070777

**Published:** 2017-07-19

**Authors:** Keiko Unno, Shigenori Noda, Yohei Kawasaki, Hiroshi Yamada, Akio Morita, Kazuaki Iguchi, Yoriyuki Nakamura

**Affiliations:** 1 Department of Neurophysiology, School of Pharmaceutical Sciences, University of Shizuoka, 52-1 Yada, Shizuoka 422-8526, Japan; iguchi@u-shizuoka-ken.ac.jp; 2 Tea Science Center, Graduate Division of Nutritional and Environmental Sciences, University of Shizuoka, Shizuoka 422-8526, Japan; yori.naka222@u-shizuoka-ken.ac.jp; 3 Division of Drug Evaluation & Informatics, School of Pharmaceutical Sciences, University of Shizuoka, Shizuoka 422-8526, Japan; s15412@u-shizuoka-ken.ac.jp (S.N.); kawasaki.yohei.2r@kyoto-u.ac.jp (Y.K.); hyamada@u-shizuoka-ken.ac.jp (H.Y.); 4 Department of Functional Plant Physiology, Faculty of Agriculture, Shizuoka University, Shizuoka 422-8529, Japan; morita.akio@shizuoka.ac.jp

**Keywords:** anti-stress effect, caffeine, green tea, middle-aged individuals, salivary α-amylase, sleep

## Abstract

Caffeine, one of the main components in green tea, can interfere with sleep and block the effect of theanine. Since theanine, the main amino acid in tea leaves, has significant anti-stress effects in animals and humans, we examined the effects of green tea with lowered caffeine content, i.e., low-caffeine green tea (LCGT), on stress and quality of sleep of middle–aged individuals (*n* = 20, mean age 51.3 ± 6.7 years) in a double-blind crossover design. Standard green tea (SGT) was used as the control. These teas (≥300 mL/day), which were eluted with room temperature water, were consumed over a period of seven days after a single washout term. The level of salivary α-amylase activity (sAA), a stress marker, was significantly lower in participants that consumed LCGT (64.7 U/mL) than in those that consumed SGT (73.9 U/mL). Sleep quality was higher in participants that consumed a larger quantity of LCGT. In addition, a self-diagnostic check for accumulated fatigue was significantly lower in those participants that consumed LCGT than SGT. These results indicate that LCGT intake can reduce stress in middle-aged individuals and improve their quality of sleep. The reduction in caffeine is suggested to be a valid reason for enhancing the anti-stress effect of green tea.

## 1. Introduction

Green tea (*Camellia sinensis* (L.) Kuntze) is the most popular drink in Japan and Asian countries. Epidemiological and animal studies have demonstrated that the ingestion of green tea enhances a healthy life [1,2]. Green tea is mainly composed of catechins (8–20%), caffeine (2–4%), and amino acids (1–8%) [3]. Epigallocatechin gallate (EGCG) is the most abundant catechin, followed by epigallocatechin (EGC). Theanine (l-theanine, *N*-ethyl-l-glutamine) is the most common amino acid, but other amino acids such as arginine (Arg) and glutamic acid (Glu) are also found in tea leaves. Some mechanisms of action of theanine, which has significant anti-stress effects in animals and humans [4,5,6,7], have been proposed, as mentioned next. Theanine, when incorporated into the brain, reportedly acts on the glutamine (Gln) transporter, and inhibits the incorporation of extracellular Gln into neurons [8]. Gln converts to Glu with the assistance of glutaminase, and Glu can be decarboxylated into γ-amino butyric acid (GABA) in neurons. In the hippocampus of mice that ingested theanine, the level of Glu was significantly reduced while the level of GABA increased [9], indicating that theanine modulates GABA production from Glu. In the brain, Glu is the main excitatory neurotransmitter while GABA is the main inhibitory neurotransmitter. Whereas suitable synaptic excitation is important, excessive excitation damages nerve cells and triggers neurodegenerative diseases [10], suggesting that the balance between Glu and GABA is important. In addition, we recently found that caffeine and EGCG suppressed the anti-stress effect of theanine while EGC and Arg retained these effects [11]. The balance between theanine, caffeine, catechins, and arginine (Arg) is suggested to be crucial for green tea to express its anti-stress effect [11]. Intervention with green tea is thought to help prevent the accumulation of stress, and to be a potential therapeutic strategy for a healthy life. However, the anti-stress effect of green tea remains unknown because it has not yet been clarified how components of green tea other than theanine influence the anti-stress effect of theanine.

Therefore, we prepared green tea with a lowered level of caffeine, low-caffeine green tea (LCGT), in which caffeine content was reduced to one-quarter to one-fifth of the level of standard green tea (SGT). Furthermore, we tried to suppress the elution of EGCG and caffeine. Since green tea is generally eluted with hot water, the eluate is rich in EGCG and caffeine. However, the solubility of EGCG and caffeine is low in room temperature water (EGCG is <5 mg/mL and caffeine is 22 mg/mL) [12]. On the other hand, the solubility of theanine is high (370 mg/mL), even at room temperature. When green tea is eluted with low temperature water, the relative ratio of theanine to EGCG and caffeine in eluate is higher than when eluted with hot water [13,14]. Therefore, in this experiment, we examined the anti-stress effect of SGT and LCGT that had been eluted with room temperature water. Indeed, the ingestion of LCGT that was steeped in room temperature water significantly suppressed the stress response in mice and in young individuals [11,12].

In this study, we examined the effect of green tea on stress responses and sleep parameters in middle-aged individuals who work in Japan. Since they drink ~1000 mL of green tea daily, drinking green tea was considered to be a basal condition. However, since there were individual differences in the amount and concentration of green tea, we requested participants to drink water for one week to washout caffeine before they consumed SGT or LCGT. In a crossover design, the effect of ingesting LCGT versus SGT was compared.

Salivary α-amylase activity (sAA), which is an oral cavity enzyme, was measured as a stress marker [15]. In humans and animals, two main systems in the body are involved in the stress response, the autonomic nervous system (ANS) and the hypothalamus-pituitary-adrenal axis. Measurement of sAA is a useful tool for monitoring ANS reactivity to stress [15]. This enzyme rapidly increases in response to physiological and psychosocial stress [16,17,18]. In addition, caffeine and a psychological stressor (20 min of mental arithmetic) are reported to increase sAA [19]. Since sAA activity is affected by sleeping time [20,21], the effects of SGT or LCGT intake on sleep parameters and the correlation between sAA levels and sleep parameters were examined. Sleep parameters were measured using a single-channel electroencephalogram (EEG). Furthermore, the effect of ingesting SGT and LCGT on subjective stress and fatigue were compared. To prevent health problems caused by overwork, the degree of fatigue accumulation in workers needs to be assessed. If ingestion of green tea can reduce chronic fatigue, then it may be considered a very useful health management tool. In this study, our objective was to examine the stress-reducing effect of green tea and to evaluate the effect of reducing the caffeine content in green tea on sAA, sleep, and subjective fatigue.

## 2. Materials and Methods

### 2.1. Preparation of LCGT

Tea leaves were collected in Kakegawa, Shizuoka, Japan. Fresh tea leaves were treated with a hot water shower at 95 °C for 180 s [11]. As a result of this treatment, caffeine was washed away from the tea leaves and at the same time the activities of the enzymes in the tea leaves were stopped. After the hot water was removed from the tea leaves by centrifugation, the tea leaves were dried through a standard manufacturing process, namely rolling and drying.

One tea bag of SGT or LCGT (3 g of tea in each bag) was steeped in 500 mL of room temperature water in a water bottle. Tap water was used in this experiment. The participants prepared SGT or LCGT every morning and ingested it until the evening. Tea bags were left in water until all tea had been fully consumed. Similarly, after each day’s work, participants drank both teas.

To measure tea components in the eluate, tea leaves of SGT or LCGT (3 g) were steeped in 500 mL of room temperature water for 0.5, 1, 3, and 6 h with occasional stirring.

### 2.2. Measurement of Tea Components by HPLC

SGT and LCGT eluates were measured by HPLC as described previously [11]. In brief, catechins and caffeine in the eluates were measured by HPLC (SCL-10Avp, Shimadzu, Kyoto, Japan; Develosil packed column ODS-HG-5, 150 × 4.6 mm, Nomura Chemical Co. Ltd., Seto, Aichi, Japan) according to the method of Horie et al. [22]. Catechins and caffeine were measured at 280 nm. Free amino acids in tea leaves were measured by HPLC as described above using homoserine as the internal standard [23]. Amino acids were detected at an excitation wavelength of 340 nm and at an emission wavelength of 450 nm using an RF-535 UV detector (Shimadzu, Japan). The relative standard deviation (RSD%) of precision and repeatability were <5.0%. The recoveries of catechins, caffeine, and free amino acids were 99 ± 4%, 98 ± 4%, and 98 ± 3%, respectively.

### 2.3. Participants

Twenty middle-aged individuals (*n* = 20, 11 male and 9 female, mean age and SD, 51.3 ± 6.7 years), who work at Kakegawa City Hall in Japan, received verbal and written information about the study and signed an informed consent form before entering the study. None of the participants indicated acute or chronic diseases, regular intake of medication, or habitual smoking. Although they drank green tea daily, they did not show symptoms of subjective insomnia due to the intake of green tea. Participants were instructed to drink mainly the test tea, and not to take theanine- and caffeine-rich beverages such as green tea, coffee, and black tea throughout the experiment. The study was conducted in accordance with the Declaration of Helsinki and Ethical Guidelines for Medical and Health Research Involving Human Subjects (Public Notice of the Ministry of Education, Culture, Sports, Science and Technology and the Ministry of Health, Labor and Welfare, 2015). The study protocol was approved by the Ethics Committee of the University of Shizuoka (No. 27-22). This study was registered at the University Hospital Medical Information Network (UMIN) (registration ID No. UMIN19411). The study period was from November to December 2015.

### 2.4. Procedure

This study was a double-blind crossover design (Table 1). Since the required sample size was calculated to be 18 participants per group, the sample size was set to 20 participants per group considering dropouts, totaling 40 participants. The participants drank water for one week to washout caffeine before they consumed LCGT or SGT. SGT was used as the control beverage. The grouping of which participants should drinking first was achieved by complete randomization. These teas (≥300 mL/day) were consumed over a period of seven days after a single washout. The primary outcome of this study was to observe the change in sAA. Sleeping hours and sAA of each participant were recorded in a questionnaire. Subjective stress at the same time every evening (~9 PM) was evaluated using visual analog scales (VAS: 0–10) from very relaxed to highly stressed and recorded in a questionnaire. The physical condition of participants was assigned an ordinal scale (5, very good; 4, good; 3, normal; 2, slightly poor; 1, poor). A self-diagnostic check for accumulated fatigue and work severity that was created by the Japanese Ministry of Health, Labor and Welfare, was carried out every Monday morning (~9 AM) and every Friday evening (~9 PM) throughout the test period. An overnight EEG was monitored for three days (from Tuesday to Thursday) in the test period.

### 2.5. Measurement of sAA

To assess the physiological stress response, sAA was measured using a testing strip, and a colorimetric system (Nipro Co., Osaka, Japan) [24]. Briefly, the testing strip consisted of a collecting strip used to collect saliva and a reagent strip used to measure sAA. A substrate in the reagent strip, 2-chloro-4-nitrophenyl-4-*O*-β-d-galactopyranosylmaltoside is hydrolyzed by sAA in the presence of maltose, a competitive inhibitor. This reaction turns the color of the reagent strip from white to yellow, and the change is quantified using a colorimetric system (sAA monitor). One unit of activity (U) per mass of enzyme is defined as the production of 1 µmol of the reducing sugar, maltose, in 1 min (NC-IUBMB, 1992).

Saliva was measured twice a day on working days: the first time was in the morning within 0.5 h after waking up (sAA before work), and the second time was in the evening before dinner (sAA after work). Prior to sampling, participants washed their mouths with water. After saliva was collected for 30 s using a testing strip, the participants measured their own sAA. The mean values among each participant and the mean values between each green tea group were compared.

### 2.6. Measurement of EEG

EEG monitoring was achieved by using a single-channel EEG (Sleep Scope, SleepWell Co., Osaka, Japan) [25], as previously described for sleep scoring programs [26,27]. The channel located at approximately Fpz-M1 was recorded for the 1ch EEG system. Before sleep, participants attached two electrodes of the Sleep Scope EEG to their forehead and mastoid to collect electrophysiological signals. Measurements started when participants entered their beds. After participants woke up and exited their beds, measurements were stopped.

The data was analyzed at the SleepWell company (SleepWell Co., Osaka, Japan) and categorized into rapid eye movement (REM) sleep and non-REM sleep, which was again classified into light sleep (N1) or slow-wave sleep (N2+N3). The onset of sleep (SL) was defined as 5 min of continuous sleep. The onset of REM (REM SL) was the time from the onset of sleep to first REM sleep. Total sleep time (TST) was calculated as the total period of sleep (SPT) minus the time spent awake during the sleep period (WASO). Sleep efficiency (SE) was the ratio of TST to the time in bed (TIB). Early morning awakening (B2 WASO) was evaluated from the time spent awake during 2 h before final awakening. Average sleep cycle (AVE SLC) is the average time from the end of REM sleep to the end of the next REM sleep. The delta wave emerges mainly during non-REM deep sleep. This value is used as an indicator of sleep depth. First delta (1st δ) is the total delta power amount during non-REM sleep in the first sleep cycle.

### 2.7. Statistical Analysis

The results are expressed as the mean ± SEM. Differences in sAA were evaluated using one-way analysis of variance (ANOVA) followed by a Tukey–Kramer post hoc test for multiple comparisons. A paired *t*-test was carried out on the differences between sAA and several sleep parameters from SGT intake to LCGT intake. All statistical analyses were carried out using Statistical Analysis System (SAS 9.4, SAS Institute Inc., Cary, NC, USA). In each analysis, a *p* value < 0.05 was considered to be statistically significant.

## 3. Results

### 3.1. Tea Components in SGT and LCGT

**Figure 1:**
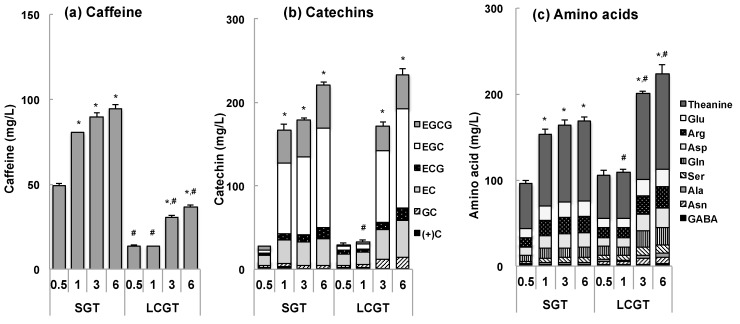
Tea components in SGT and LCGT. One tea bag of SGT or LCGT (3 g of tea in a bag) was steeped in 500 mL of room temperature water (0.5–6 h). Tap water was used in this experiment. Data are expressed as mean ± SD (*n* = 3). The data of SGT or LCGT was compared with that at 0.5 h, respectively (*, *p* < 0.05). In addition, LCGT data was compared with SGT data at the same elution time (#, *p* < 0.05).

The content of caffeine in LCGT steeped in room temperature water was about 0.3~0.4 times that of SGT (Figure 1a). Although EGCG was the main catechin in tea leaves, EGC was the most abundant catechin in the eluate with room temperature water (Figure 1b). The total amount of catechins was almost the same between SGT and LCGT at all elution times, except 1 h. The amount of theanine in LCGT was about 1.2 times higher than in SGT (Figure 1c). Arg in LCGT was about 1.4 times higher than in SGT. The total amount of amino acids was 1.3 times higher in LCGT than in SGT after elution for 3 and 6 h. The concentration of each component increased with elution period, but the relative ratio was almost the same. 

### 3.2. Interaction between sAA Level and LCGT Intake

**Figure 2:**
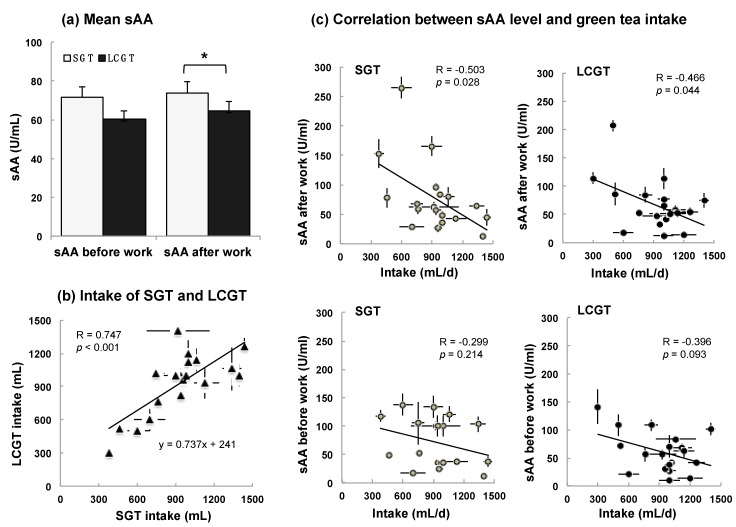
Anti-stress effect of LCGT. (**a**) Mean sAA level of each group. Data are expressed as mean ± SEM (*n* = 19, *, *p* < 0.05; one-way ANOVA); (**b**) Correlation between SGT and LCGT intake; (**c**) Correlation between the level of sAA before work or after work and SGT or LCGT intake. Data of b and c are expressed as mean ± SEM (*n* = 5, in each participant).

Among the 20 initial participants, one abstained due to poor physical conditions. Nineteen participants drank SGT or LCGT every day from Saturday to Friday. During working days, the participants measured their own level of sAA every morning and evening—i.e., before work and after work, respectively—and, in the evening, recorded the volume consumed throughout the day. The mean levels of sAA in all participants were compared between the SGT and LCGT groups. The level of sAA after work was significantly lower when participants consumed LCGT than when they consumed SGT (Figure 2a, *p* = 0.043). Similarly, the level of sAA before work tended to be lower in the LCGT group than in the SGT group (*p* = 0.109).

The mean volumes consumed by each participant were 929 ± 31 mL of SGT and 927 ± 34 mL LCGT. LCGT intake was closely correlated with SGT intake in each participant (Figure 2b, *p* < 0.001). Then, the interaction between sAA level and ingestion volume was examined. Ingestion volume was negatively and significantly correlated with the level of sAA after work (Figure 2c, SGT, *p* = 0.028; LCGT, *p* = 0.044). The correlation between ingestion volume and the levels of sAA before work was low (Figure 2c, SGT, *p* = 0.214; LCGT, *p* = 0.093).

**Figure 3:**
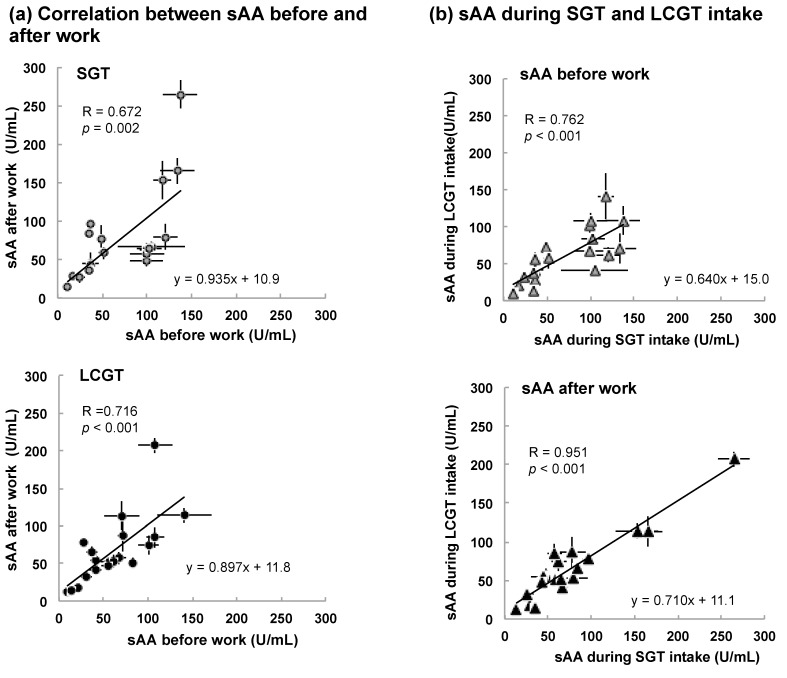
Level of sAA in each participant. (**a**) Correlation between sAA before and after work; (**b**) Correlation of sAA before or after work between during SGT and LCGT intake. Data are expressed as mean ± SEM (*n* = 5, in each participant).

Although there were individual differences in the levels of sAA, participants with higher sAA before work showed higher sAA after work in both SGT and LCGT groups (Figure 3a, SGT *p* = 0.002, LCGT *p* < 0.001). There was no significant difference in the slope of the approximate line (SGT 0.935, LCGT 0.897). Then, the effect of SGT and LCGT intake was compared on the level of sAA in each participant. The levels of sAA became low in each participant by changing from SGT to LCGT (Figure 3b, the slope of the approximate line; sAA before work 0.640, sAA after work 0.710).

### 3.3. Effect of SGT and LCGT Ingestion on Sleep Parameters

**Figure 4:**
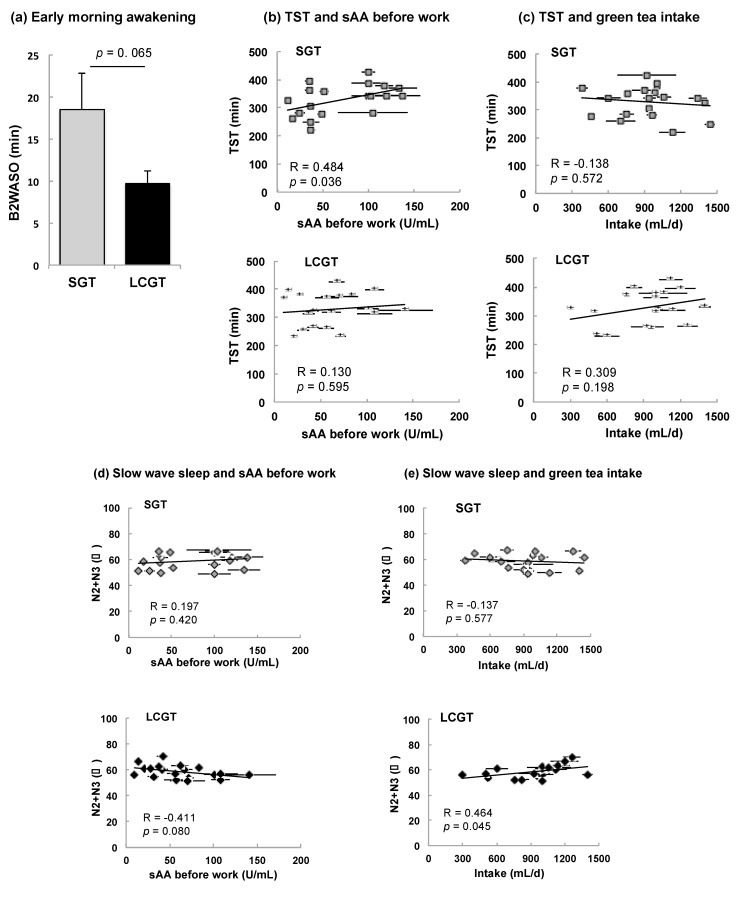
Effect of green tea intake on sleep parameters or sAA. (**a**) Early morning awakening. Data are expressed as mean ± SEM (*n* = 19, *, *p* = 0.065; one-way ANOVA); (**b**) Correlation between TST and sAA before work; (**c**) Correlation between TST and intake volume; (**d**) Correlation between slow wave sleep (N2+N3) and sAA before work; (**e**) Correlation between slow wave sleep and intake of SGT or LCGT. Data of b–e are expressed as mean ± SEM (*n* = 5, in each participant).

Sleep data of the participants was obtained using a 1ch EEG system. Since there was no difference in sleep time of each participant in the period of EEG measurements, data from the second day was used for the analysis. The data of sleep parameters when each participant drank SGT or LCGT are shown in Table 2. Mean values of SL, REM SL, SPT, TST, REM, N1, N2+N3, WASO, AVR SLC, SE, and 1st δ did not differ significantly between SGT and LCGT. However, the time of B2 WASO tended to be shorter when participants ingested LCGT than SGT (Table 2 and Figure 4a, *p* = 0.065). The level of sAA before work was closely correlated with SPT and TST in participants that ingested SGT (Table 2 and Figure 4b, *p* = 0.046 and 0.036, respectively). There was no correlation between TST and SGT intake (Figure 4c). The level of N2+N3 tended to be negatively correlated with sAA before work in the LCGT group (Figure 4d, *p* = 0.080). A positive correlation was observed between intake volumes of LCGT and the level of N2+N3 (Figure 4e, *p* = 0.045). There was no significant relationship between sAA after work and sleep parameters (Table 2).

### 3.4. Subjective Stress and Fatigue

**Figure 5:**
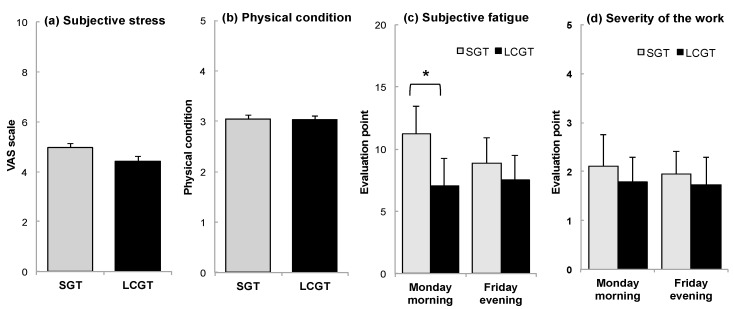
Effect of green tea ingestion on subjective stress and fatigue. (**a**) Subjective stress; (**b**) physical condition; (**c**) subjective fatigue; and (**d**) severity of work. Data are expressed as mean ± SEM (*n* = 19, *, *p* < 0.05; one-way ANOVA).

Subjective stress tended to be lower when participants drank LCGT than SGT (Figure 5a, *p* = 0.152), although the physical condition between LCGT and SGT groups was not different (Figure 5b, *p* = 0.779). The accumulated fatigue that participants felt on Monday morning was significantly lower when they drank LCGT (Figure 5c, *p* = 0.015). Subjective fatigue on Friday evening tended to be lower in the LCGT group than the SGT group (Figure 5c, *p* = 0.074). The evaluation of work severity was similar when participants drank LCGT or SGT (Figure 4d, Monday *p* = 0.316, Friday *p* = 0.531).

## 4. Discussion

The level of sAA after work was significantly lower in the LCGT group than in the SGT group (Figure 2a). The main difference between LCGT and SGT was the amount of caffeine (Figure 1a). Caffeine intake was ~1 mg/kg in those participants who ingested LCGT. Low dose of caffeine (0.32 mg/kg) did not suppress stress response in mice, but was canceled by theanine [11]. In addition, EGCG suppressed the anti-stress effect of theanine [11]. Thus, low doses of caffeine are not beneficial, but suggest that the interaction among theanine, caffeine, and EGCG is important for suppression of stress. In SGT, the ratio of theanine: caffeine: EGCG was 1:1:0.5 while in LCGT, it was 1:0.3:0.3. Since the ratio of theanine to caffeine and EGCG was 2.5 times higher in LCGT than in SGT, the anti-stress effect of theanine was considered to be higher in LCGT than SGT. 

Participants that ingested <600 mL of LCGC exhibited a higher level of sAA than the mean (64.7 U/mL) (Figure 2c). That is, intake of at least 600 mL of LCGT may be required to suppress sAA. When LCGT was eluted with room temperature water, in the case of 3 h elution, the concentration of theanine was 100 mg/L (Figure 1c). It can be extrapolated that at least 60 mg/day of theanine may be needed to suppress stress. A similar anti-stress effect had been observed in elderly participants that ingested ≥60 mg/day of theanine from LCGT [28].

Whereas there was no significant difference in the mean value of various sleep parameters between SGT and LCGT, the early morning awakening time tended to be shorter by changing from SGT to LCGT. Early morning awakening is a frequent complaint among patients with sleep disorders [29,30]. The ratio of non-REM sleep (N2+N3), a marker of deep sleep, was significantly higher in those participants that consumed a higher amount of LCGT. Increased theanine intake may increase sleep quality. The time of SPT and TST was closely correlated with sAA before work rather than after work, suggesting that sAA after work does not influence sleep, but sleep affects sAA before work. Participants of high sAA before work may have required a longer sleeping time. Indeed, the average sleeping time of participants was short (5.5 h).

Subjective fatigue is reported to be higher on Monday than on Friday [31]. It is worth noting that subjective fatigue was significantly reduced by LCGT intake. High subjective fatigue on Monday is greatly affected by weekend rest and lack of motivation at the beginning of the week [31]. The levels of sAA were not measured on the weekend; however, the ingestion of green tea was initiated from Saturday. Therefore, participants in the LCGT group are presumed to have lower sAA than members of the SGT group.

In workers who habitually have a short sleep pattern (mean weekday sleep ≤6 h), the extension of sleep on weekends improves alertness and performance during the first few days in subsequent weeks [32]. Strategic naps may reduce subjective feelings of fatigue and improve performance and alertness [33]. The participants in this study had a habitually short amount of sleep, but sleep extension on the weekend and naps were not observed (data not shown). This suggests that the suppression of stress on the weekend is important for adequate rest on the weekend and to stimulate high motivation at the beginning of the week.

We aimed to explore the effect of green tea with reduced caffeine content on the stress of middle-aged individuals. However, this study has several limitations. Firstly, this study has a small number of participants. However, the sAA analyses using individual differences complemented the small number of participants. Secondly, SGT was used as a control beverage, because water was not a suitable placebo in the blind test. Since participants drank green tea daily, to withdraw the effect of caffeine, a seven-day washout period was set before the test term [34]. Then, intake of LCGT was compared with that of SGT. Thirdly, we used a single-channel EEG instead of gold–standard polysomnography (PLG) to assess sleep. However, it has recently been shown that a single-channel EEG can also be a useful research tool in assessing REM, non-REM sleep and several other parameters [35]. Fourthly, we did not measure the components of all the tea samples that were prepared daily by participants. One possible problem is that the concentration of tea components among participants may have been different. However, the eluate data indicates that the ratio of catechins, caffeine, and amino acids was almost the same in SGT or LCGT (Figure 1), suggesting that the content of tea components was similar under the same elution conditions, including the same volume of tea leaves and water, and a similar temperature. Therefore, despite these limitations, the anti-stress effect of green tea obtained in this experiment is considered to be reliable.

## 5. Conclusions

The effects of LCGT on stress and sleep were examined in middle–aged individuals (*n* = 19, mean age 51.3 ± 6.7 years) in a double-blind crossover design using SGT as the control beverage. The level of sAA, a maker of stress, was significantly lower in the participants that consumed LCGT than SGT. Furthermore, ingestion of LCGT significantly improved sleep quality and reduced subjective fatigue on Monday morning. These results suggest that a reduction of caffeine in green tea is beneficial for reducing stress. Simultaneously with the lowering of caffeine, the increase in theanine and Arg are considered to be necessary, it is necessary to further clarify the mechanism of action of the stress-reducing effect of green tea components. 

## Figures and Tables

**Table 1:** Experimental protocol.

Term (1 Term with 7 Days)	1	2	3	4
Green tea intake	Washout (water)	SGT or LCGT	Washout (water)	LCGT or SGT
	(Saturday~Friday)	(Saturday~Friday)	(Saturday~Friday)	(Saturday~Friday)
Measurement of α-amylase activity	(-)	2 times/day (morning and evening)	(-)	2 times/day (morning and evening)
Subjective stress and Physical condition	(-)	every evening	(-)	every evening
Sleep	(-)	3 nights/week (Tuesday~Thursday)	(-)	3 nights/week (Tuesday~Thursday)
Subjective fatigue	(-)	2 times/week (Mon morning and Fri evening)	(-)	2 times/week (Monday morning and Friday evening)

This study was conducted in a double-blind crossover design. Participants drank SGT or LCGT that was eluted with room temperature water for a period of seven days after a single washout with water.

**Table 2:** Mean value of each sleep parameter and the correlation between sleep parameters and sAA or intake volume when participants drank SGT or LCGT.

Sleep Parameters	Abbreviation	Standard Green Tea							Low-Caffeine Green Tea						
		Mean ± SEM	Correlation between sAA before Work		Correlation between sAA after Work		Correlation between Intake Volume		Mean ± SEM	Correlation between sAA before Work		Correlation between sAA after Work		Correlation between Intake Volume	
			R	*p* Value	R	*p* Value	R	*p* Value		R	*p* Value	R	*p* Value	R	*p* Value
Onset of sleep (min)	SL	12.6 ± 3.0	−0.129	0.599	−0.239	0.325	0.356	0.135	15.7 ± 4.9	0.075	0.759	−0.026	0.915	−0.138	0.572
Onset of REM (min)	REM SL	63.5 ± 5.3	0.184	0.450	−0.199	0.415	0.427	0.068	57.7 ± 3.0	0.045	0.854	−0.045	0.854	0.304	0.207
Total period of sleep (min)	SPT	351.3 ± 12.5	0.462	0.046 *	0.232	0.339	−0.136	0.579	350.2 ± 14.6	0.173	0.479	0.024	0.921	0.301	0.211
Total sleep time (SPT−WASO) (min)	TST	328.7 ± 12.7	0.484	0.036 *	0.2672	0.269	−0.138	0.572	328.6 ± 13.5	0.130	0.595	0.040	0.872	0.309	0.198
Rapid eye movement (%)	REM	28.2 ± 2.5	0.066	0.787	−0.082	0.739	−0.106	0.665	25.9 ± 1.2	0.077	0.753	0.142	0.562	−0.186	0.446
Light sleep (%)	N1	8.7 ± 0.7	0.361	0.129	0.2039	0.402	−0.055	0.822	9.3 ± 0.8	0.285	0.236	0.375	0.113	−0.438	0.061
Slow wave sleep (%)	N2+N3	58.8 ± 1.4	0.197	0.420	0.1261	0.607	−0.137	0.577	58.7 ± 1.2	−0.411	0.080	−0.351	0.141	0.464	0.045 *
Time spent awake during the sleep (min)	WASO	22.6 ± 2.9	−0.117	0.635	−0.163	0.505	0.018	0.943	21.7 ± 2.3	0.329	0.169	−0.076	0.757	0.096	0.697
Total awakening time during two hours before the final awakening (min)	B2 WASO	18.5 ± 4.4	0.009	0.969	−0.219	0.367	−0.126	0.607	9.7 ± 1.5	−0.018	0.941	−0.139	0.571	0.250	0.301
Average sleep cycle (min)	AVR SLC	91.5 ± 3.2	−0.032	0.895	−0.268	0.267	−0.189	0.437	90.4 ± 2.9	0.006	0.979	−0.248	0.306	0.176	0.471
Sleep eficiency (%)	SE	88.5 ± 1.7	0.099	0.688	0.281	0.244	−0.122	0.620	89.2 ± 1.5	−0.153	0.533	0.055	0.824	0.110	0.654
δ power in the 1st sleep cycle (µV^2^)	1st δ	149,256 ± 18,656	−0.031	0.900	−0.157	0.520	−0.052	0.832	120644 ± 15923	−0.019	0.938	0.158	0.518	−0.254	0.295
sAA (U/mL)			71.7 ± 5.2		73.9 ± 5.9					60.5 ± 4.3		64.7 ± 4.8			
Intake volume (mL)							929 ± 31							927 ± 34	

Data of sleep parameters are expressed as mean ± SEM (*n* = 19). Correlation coefficient (R) and *p* value between each sleep parameter and sAA or intake volume of each participant are expressed (*, *p* < 0.05).
